# Supplementary material for: Exploratory Transcriptomic Profiling Reveals the Role of Gut Microbiota in Vascular Dementia
Source: Int J Mol Sci. 2023 Apr 30;24(9):8091. doi: 10.3390/ijms24098091 (PMC10178712; doi:10.3390/ijms24098091)
Supplement: Supplementary file 1 [file ijms-24-08091-s001.zip › ijms-2341488-supplementary.pdf]

Exploratory Transcriptomic Profiling Reveals the Role of Gut Microbiota in Vascular Dementia

Navdeep Kaur [1], Geneva LaForce [1], Deepthi P. Mallela [1], Prasenjit Prasad Saha [1], Jennifer Buffa [1], Xinmin S. Li [1], Naseer Sangwan [1,2], Kasia Rothenberg [3] and Weifei Zhu [1,*]

[1] Department of Cardiovascular & Metabolic Sciences, Cleveland Clinic, Cleveland, OH 44195, USA; kaurn7@ccf.org (N.K.); laforcg@ccf.org (G.L.); malleld@ccf.org (D.P.M.); sahap2@ccf.org (P.P.S.); buffaj@ccf.org (J.B.); lix2@ccf.org (X.S.L.); sangwan@ccf.org (N.S.)
[2] Microbial Sequencing & Analytics Resource (MSAAR) Facility, Shared Laboratory Resources (SLR), Lerner Research Institute, Cleveland Clinic, Cleveland, OH 44195, USA
[3] Cleveland Clinic Lou Ruvo Center for Brain Health, Cleveland, OH 44195, USA; rothenk@ccf.org
* Correspondence: zhuw@ccf.org; Tel.: +1-216-445-0990

Table S1: Differentially expressed genes with highest fold change (up/down) in left brain cortex of mice in choline and control groups.

Pre								***Day 3***							
**Upregulated**				**Down-regulated**				**Upregulated**				**Down-regulated**			
**symbol**	**entrez**	**logfc**	**adjpv**	**symbol**	**entrez**	**logfc**	**adjpv**	**symbol**	**entrez**	**logfc**	**adjpv**	**symbol**	**entrez**	**logfc**	**adjpv**
Cyp2a5	13087	2.404416	0.002041	Prl	19109	-4.353168	0.002926	Ifi206	1.03E+08	4.288259	0.000432	Pou4f2	18997	-6.17803	0.001996
Wnt9b	22412	1.959597	0.004405	Gh	14599	-4.2244	0.005778	Lcn2	16819	4.262434	0.000001	Gata3	14462	-4.69422	0.000576
Gfy	100039953	1.881408	0.005606					Cd5l	11801	4.03055	0.000281	Sox14	20669	-4.66157	0.001471
Day 1								Phf11a	219131	3.677417	0.003795	Pax7	18509	-3.86859	0.006424
Tgm1	21816	4.577848	0.00025	Hcrt	15171	-3.329184	0.028934	Saa3	20210	3.429372	0.006763	Barhl1	54422	-3.71657	0.001745
Mmp3	17392	4.390026	0.00018	Npas4	225872	-2.405589	0.000171	Cga	12640	3.346448	0.036771	Gm5903	546005	-3.03644	0.009947
Slfn4	20558	3.931423	0.00052	Gm28707	1.08E+08	-2.180096	0.013786	Scgb1c1	338417	3.298835	0.014407	Irx2	16372	-2.97009	0.004798
Pax7	18509	3.726796	0.028461	Lrat	79235	-1.991012	0.029157	Ccl4	20303	3.288576	0.00017	Rny3	19874	-2.82025	0.018581
Lilr4b	14727	3.707754	7.14E-05	Lrrc14b	432779	-1.884303	0.02135	Cxcl10	15945	3.2142	0.00012	Gm26795	1.05E+08	-2.79683	0.006767
Krt75	109052	3.694855	0.000281	Snord53	1E+08	-1.813964	0.00539	Gh	14599	2.992638	0.034273	Shox2	20429	-2.77205	0.002132
Barhl1	54422	3.682261	0.006382	Depp1	213393	-1.780384	0.00105	Cxcr2	12765	2.905243	0.013676	Snord59a	1.15E+08	-2.6818	0.036974
Rbp3	19661	3.681021	0.001367	Wnt8b	22423	-1.649532	0.031914	Afp	11576	2.868992	0.001129	Smpx	66106	-2.66251	0.030002
Ptx3	19288	3.674518	0.000563					Cyp4f18	72054	2.795326	0.000366	Gm12394	1.01E+08	-2.62272	0.000156
ETV3L	546801	3.580271	0.001903					Prl	19109	2.786142	0.044138	Dmbx1	140477	-2.55422	0.003393
Ccl2	20296	3.564794	0.00025					Lilr4b	14727	2.745565	0.000843	Cer1	12622	-2.51328	0.002629
Cxcl1	14825	3.290339	5.09E-05					Slfn4	20558	2.71014	0.007577	Lhx1	16869	-2.45098	0.005104
Crct1	74175	3.257868	0.001135					Ifi204	15951	2.692151	4.63E-06	Gm25939	1.15E+08	-2.377	0.046339
Ccl12	20293	3.23896	0.000294					Cd300lf	246746	2.687131	0.000148	Ckm	12715	-2.34652	0.045615
Cxcl10	15945	3.161448	0.000684					Ifi211	381308	2.668883	0.003465	Rny1	19872	-2.22011	0.0027
Npw	381073	3.150283	0.000448					Tgm1	21816	2.665939	0.023581	Gm5237	383326	-2.14491	0.016225
Hcar2	80885	2.870404	0.008091					Gm33370	1.03E+08	2.658115	0.00921	Gm24978	1.15E+08	-2.13372	0.008985
Apol9a	223672	2.799433	0.012805					Ifi209	236312	2.651839	0.000267	Atp2a1	11937	-2.11256	0.000266
Gata3	14462	2.795749	0.03508					Mmp3	17392	2.640886	0.02582	Gm48249	1.15E+08	-2.07508	0.005051
Serpinb5	20724	2.738153	0.000247					Mx1	17857	2.595755	0.00012	Rbp3	19661	-2.05321	0.032884
Msr1	20288	2.706369	5.2E-05					Zbp1	58203	2.543091	0.000149	Foxb1	64290	-2.01172	0.045135
Lif	16878	2.689888	0.000284					Ifi27l2a	76933	2.526809	0.000001	Wnt9b	22412	-1.97826	0.001718
Fpr2	14289	2.54672	0.006344					Ccl12	20293	2.497871	0.002881	Hs6st3	50787	-1.96594	0.000001

Cryba2	12958	2.535041	1.94E-05					Gm12297	668323	2.493415	0.017839	Pcdha1	116731	-1.9557	0.000001
Fam83g	69640	2.50539	0.000395					AI662270	1E+08	2.489103	0.001876	Gm38058	1.05E+08	-1.95349	0.001371
Il11	16156	2.498481	5.24E-06					Msr1	20288	2.477537	5.48E-05	Tpd52-ps	107730	-1.92201	0.023478
Mmp12	17381	2.488482	0.03701					Isg15	1E+08	2.449203	3.42E-06	Gm46563	1.08E+08	-1.9102	0.014768
Cst7	13011	2.488404	0.005434					Arg1	11846	2.447362	0.001583	Gm21297	1.01E+08	-1.89136	0.017134
Bdkrb2	12062	2.485501	0.000001					Clec4d	17474	2.446059	0.046051	Gm10010	434089	-1.83031	0.000001
Ccl7	20306	2.465872	0.006233					Ccl8	20307	2.411758	0.000853	Myh4	17884	-1.81042	7.41E-05
Cd44	12505	2.449625	5.38E-06					Ifi207	226691	2.409409	2.6E-06	Pik3c2g	18705	-1.78674	0.009965
P2ry2	18442	2.434637	0.000001					Plac8	231507	2.408942	0.003606	Gm15462	1.08E+08	-1.78329	0.020651
Cd244a	18106	2.418923	0.011008					BC023105	667597	2.398616	0.016846	Gm5830	545332	-1.74723	0.033144
Milr1	380732	2.404962	0.000718					Glycam1	14663	2.392136	0.001993	Glra1	14654	-1.6423	0.020232
Serpinf2	18816	2.38817	0.000553					Apol9a	223672	2.392033	0.003152	Kcnh5	238271	-1.63881	0.000001
Clcf1	56708	2.364962	0.000001					Gm12104	1E+08	2.384326	0.044887	Ush2a	22283	-1.60327	0.025735
Timp1	21857	2.348033	0.002202					Ptx3	19288	2.379121	0.021704	Gm26560	1.03E+08	-1.60229	0.03539
S100a8	20201	2.335234	7.09E-05					9330175E14Rik	320377	2.375727	0.000147	Exph5	320051	-1.60224	0.000001
Gdf15	23886	2.325783	0.000893					Oas1g	23960	2.375218	0.00023	Pakap	677884	-1.59749	0.000001
Tmem40	94346	2.249585	0.000001					Cd300ld	217305	2.356666	0.009142	Gm12462	1.01E+08	-1.59009	0.007753
Gja3	14611	2.199436	9.15E-05					Lilrb4a	14728	2.355949	0.000128	Tfap2b	21419	-1.58273	0.003268
Tubb6	67951	2.184872	0.001652					Clec7a	56644	2.352357	0.000001	6330411D24Rik	70725	-1.56637	0.036994
Bcl3	12051	2.172518	0.000555					Gm4841	225594	2.350735	0.045424	Chrna6	11440	-1.56331	0.006304
Cd300lf	246746	2.168166	0.012073					2700078F05Rik	72626	2.321723	0.012689	Gm6430	623430	-1.55447	0.038675
Loxhd1	240411	2.165585	5.25E-05					S100a4	20198	2.304241	0.0144	Gm15723	1.03E+08	-1.54713	6.24E-05
Klk2-ps	16603	2.14369	0.005589					Phf11b	236451	2.302384	0.000119	Slc9a7	236727	-1.54662	0.000001
Lilrb4a	14728	2.141679	0.001697					Fcgr4	246256	2.296147	0.001213	Nt5c1a	230718	-1.54213	0.000001
Wnt9b	22412	2.131315	0.000337					Ccl3	20302	2.27883	0.001347	Gdf7	238057	-1.53564	0.011957
Emp1	13730	2.116216	1.51E-06					Ms4a4c	64380	2.276792	0.006767				
Serpine1	18787	2.089896	0.00011					AI427809	381524	2.273888	0.007515				
Has2	15117	2.077019	0.001022					Cxcl1	14825	2.270783	0.00537				

Drd4	13491	2.073883	0.007889					Gm36738	1.03E+08	2.261474	0.003305				
Selp	20344	2.059762	0.030245					Tspan32	27027	2.245872	0.000109				
Rufy4	435626	2.055115	0.011455					Timp1	21857	2.211824	0.001729				
Gm6477	624165	2.045394	0.007995					Cd244a	18106	2.186262	0.011451				
Helt	234219	2.011196	0.001121					Osm	18413	2.183298	0.007431				
Socs3	12702	2.009069	6.13E-06					Lvrn	74574	2.17465	0.040951				
Flnc	68794	2.001892	0.000846					Ifi213	623121	2.147552	0.000392				
Epcam	17075	1.951101	0.007503					Cd52	23833	2.145765	0.000001				
Glrp1	14659	1.942994	0.048898					Ska1	66468	2.145742	0.001431				
Oasl1	231655	1.942736	0.009746					Ms4a6c	73656	2.103491	0.000001				
Spp1	20750	1.937613	0.000001					Gm12250	631323	2.087984	8.77E-05				
Gm10146	100041500	1.905186	0.048109					Oas3	246727	2.082936	0.008599				
Areg	11839	1.896804	0.03155					Ccl2	20296	2.08049	0.025697				
Cd28	12487	1.873203	0.000838					1810073O08Rik	72285	2.065749	0.008838				
Fndc9	320116	1.871577	1.1E-05					Trim30b	244183	2.060661	0.000169				
Ppm1j	71887	1.870028	0.0005					Hcar2	80885	2.049968	0.040129				
Fndc7	320181	1.845089	0.009748					H19	14955	2.04641	0.000153				
Usp18	24110	1.841907	0.001724					2900076G11Rik	73052	2.046095	0.006892				
Tnn	329278	1.833778	0.000323					Padi1	18599	2.040353	0.030987				
Gm4951	240327	1.82468	0.005563					A930001C03Rik	319314	2.039156	0.025343				
Gm10791	100038614	1.820468	0.001952					Usp18	24110	2.038324	8.16E-05				
Tnfrsf12a	27279	1.786689	5.59E-05					Cyp21a2-ps	632302	2.037824	0.016326				
S100a9	20202	1.775071	0.010382					Chil3	12655	2.03237	0.004846				
6430628N08Rik	76237	1.761512	0.00026					Pilra	231805	2.025128	0.000229				
Ccl4	20303	1.753613	0.043986					Ddx60	234311	2.016919	0.000001				
Gldn	235379	1.74923	2.29E-06					Mcoln2	68279	2.014875	0.042477				
Ifi204	15951	1.746395	0.017245					Tnfrsf26	244237	2.010884	0.049942				

Zbp1	58203	1.726513	0.030282					Gm26739	1.03E+08	2.002909	0.046091				
Hspb1	15507	1.707225	0.000001					Gm1966	434223	1.99918	0.000783				
Gbp6	100702	1.701746	0.000922					Aoc1	76507	1.990454	0.045912				
Gm4070	100042856	1.699077	0.005437					Plxna4os1	319849	1.979057	1.16E-05				
Chil3	12655	1.690709	0.04551					C3	12266	1.976125	4.4E-06				
Tnfaip2	21928	1.685262	8.05E-05					Ifi44	99899	1.971615	0.000159				
9330175E14Rik	320377	1.681914	0.018227					Ly9	17085	1.965365	0.000001				
AA467197	433470	1.675375	0.013002					Gm4951	240327	1.96453	0.000279				
Col27a1	373864	1.674806	0.000001					Pkp3	56460	1.963734	0.043439				
Ifi207	226691	1.668717	0.007086					Cdk1	12534	1.963525	1.86E-05				
Gimap4	107526	1.658516	0.000494					Cxcl5	20311	1.955344	0.002315				
Mx1	17857	1.65505	0.042782					Oasl1	231655	1.954119	0.001642				
Dnmt3l	54427	1.651336	0.034793					Gm24608	1.15E+08	1.950373	0.046876				
Rsad2	58185	1.649832	0.000639					Oas1a	246730	1.949236	1.56E-05				
Ccr1	12768	1.639494	0.023172					Ifi208	1E+08	1.94682	0.033539				
Lgals3	16854	1.635513	0.003085					Aurkb	20877	1.940512	4.64E-05				
Adam5	11499	1.634262	0.042272					Tk1	21877	1.936247	1.75E-06				
Rgs13	246709	1.616832	0.000207					Irf7	54123	1.932717	6.09E-06				
Akr1b8	14187	1.607128	0.000294					Ccl5	20304	1.92346	0.026922				
Sdc1	20969	1.607033	6.24E-05					Ankle1	234396	1.910549	7.08E-05				
H19	14955	1.596965	0.009774					Neil3	234258	1.910236	0.00022				
Mcemp1	69189	1.568241	0.009578					4930474N09Rik	67639	1.895032	0.032749				
Slamf8	74748	1.553444	0.035113					Phf11d	219132	1.892799	8.18E-06				
Ntf5	78405	1.552736	0.008961					Milr1	380732	1.881361	0.000246				
Atp2a1	11937	1.55229	0.023507					F830016B08Rik	240328	1.879675	0.012562				
Lpo	76113	1.551631	0.00036					1600010M07Rik	69781	1.86977	0.011053				
Gfap	14580	1.549129	0.000908					Il18rap	16174	1.868187	0.00796				
Gm24339	115487283	1.544039	0.046317					S100a9	20202	1.867289	0.00404				

Plaur	18793	1.537554	6.96E-05					Bik	12124	1.866859	0.035038				
F2rl2	14064	1.537444	2.07E-05					Bst2	69550	1.861487	0.000001				
Tafa3	329731	1.536897	0.036677					Lgals3	16854	1.850953	0.000139				
Clec7a	56644	1.530771	0.002262					Apob	238055	1.848731	0.030962				
Fgr	14191	1.529849	0.041384					Pbk	52033	1.845922	4.44E-05				
A730043L09Rik	330958	1.518965	0.015612					Cd300lb	217304	1.84569	0.047395				
Lox	16948	1.514877	0.001251					Oas2	246728	1.844642	3.44E-05				
Hbegf	15200	1.511315	2.03E-06					Ifit3	15959	1.826746	0.000001				
Snai3	30927	1.503418	0.001085					Gm6581	625409	1.821235	0.046501				
Il1b	16176	1.502793	0.003321					Gm19209	1E+08	1.818946	0.006663				
								Spp1	20750	1.812654	0.000001				
								Rtp4	67775	1.807071	0.000001				
								Cst7	13011	1.806014	0.006315				
								Siglec1	20612	1.805287	0.000277				
								Gm10862	1E+08	1.802859	0.006836				
								Gm553	227091	1.785662	0.039668				
								Icos	54167	1.772254	0.010965				
								Gm15327	1.01E+08	1.768917	0.033681				
								B430306N03Rik	320148	1.768147	0.000472				
								Itgax	16411	1.752233	9.69E-06				
								Bcl3	12051	1.750536	0.003465				
								Ifi30	65972	1.740273	0.000001				
								Klk6	19144	1.739374	0.000001				
								AA467197	433470	1.738767	0.001724				
								Oas1b	23961	1.730292	6.31E-05				
								Ube2c	68612	1.724392	0.000252				
								Tlr2	24088	1.718451	0.000001				
								Tnfsf8	21949	1.717056	0.040074				
								Cdca5	67849	1.71683	0.018842				

								Ccr1	12768	1.716309	0.005811				
								Nlrc5	434341	1.705795	0.000001				
								Steap4	117167	1.704135	0.000196				
								Mx2	17858	1.70226	2.26E-06				
								Pirb	18733	1.689721	1.37E-05				
								Vim	22352	1.687308	1.66E-05				
								Dyrk4	101320	1.685305	0.00136				
								Ms4a6d	68774	1.679912	0.000001				
								Ifit3b	667370	1.669798	2.15E-06				
								Slfn9	237886	1.667366	0.000458				
								Batf	53314	1.662503	0.001373				
								Serpina3n	20716	1.661562	7.34E-05				
								Slamf6	30925	1.659072	4.25E-05				
								Mmp13	17386	1.652609	0.036547				
								Cybb	13058	1.647281	6.14E-05				
								Pimreg	109212	1.645763	0.001284				
								Uhrf1	18140	1.645173	0.000001				
								Oasl2	23962	1.645147	6.56E-05				
								Slamf8	74748	1.634947	0.019128				
								Ifitm3	66141	1.634034	0.000001				
								Iigp1	60440	1.629524	2.91E-05				
								Arl11	219144	1.627967	0.000001				
								Dhh	13363	1.627165	0.030688				
								Ppp1r18os	1.01E+08	1.626003	0.012356				
								Epsti1	108670	1.617884	0.000001				
								Ch25h	12642	1.61737	1.43E-06				
								S100a8	20201	1.610968	0.004297				
								Plbd1	66857	1.609225	0.006716				
								Sap30bpos	1.01E+08	1.60453	0.000111				
								Psmb8	16913	1.604049	0.000001				

								Birc5	11799	1.598554	0.000001				
								Gpr65	14744	1.588515	0.005436				
								Cd72	12517	1.583529	0.00011				
								Pclaf	68026	1.583471	0.00075				
								Gm26564	1.03E+08	1.575443	0.014209				
								Treml2	328833	1.575044	0.000473				
								Gm12185	620913	1.57489	0.013055				
								Fcgr1	14129	1.555369	3.94E-06				
								S100a6	20200	1.54973	0.000001				
								Casp4	12363	1.544363	2.26E-05				
								Trim30a	20128	1.5442	0.000001				
								Hp	15439	1.543442	0.028784				
								Troap	78733	1.540657	0.000493				
								F630028O10Rik	1E+08	1.539632	0.002727				
								Rsad2	58185	1.539328	0.000275				
								Gbp8	76074	1.538115	0.012018				
								Gbp3	55932	1.537651	0.000001				
								Ms4a14	383435	1.53741	0.009656				
								Cxcl16	66102	1.536425	0.000001				
								BC147527	625360	1.532775	0.044774				
								Gpr84	80910	1.532571	5.8E-05				
								Gm15808	1E+08	1.527257	0.029173				
								Cep55	74107	1.525596	0.000588				
								Cd22	12483	1.518063	0.001027				
								C5ar1	12273	1.51763	7.14E-05				
								Gm12530	329851	1.510832	0.008588				
								AU022793	105976	1.508559	0.018112				
								C3ar1	12267	1.507408	0.000001				

								Sgo1	72415	1.505925	0.00055				
								Ly6g5b	266614	1.504451	0.009235				
								Gbp6	100702	1.501921	0.000413				

Table S2: The top 5 gene ontology (GO) terms of the differentially expressed genes detected in the ischemic mouse brain at pre, and post-stroke days 1 and 3.

Pre		Day 1		Day 3	
GO Term	p-value	GO Term	p-value	GO Term	p-value
Biological Processes					
Not significant		biological regulation	0.005	cellular process	2.498e-4
		regulation of biological process	0.003	biological regulation	1.701e-12
		regulation of cellular process	0.003	regulation of biological process	1.264e-14
		response to stimulus	1.155e-18	regulation of cellular process	2.072e-11
		multicellular organismal process	1.080e-11	response to stimulus	1.000e-24
Molecular Functions					
signaling receptor regulator activity	0.036	protein binding	0.001	binding	1.490e-4
signaling receptor activator activity	0.021	signaling receptor binding	6.344e-12	protein binding	4.470e-8
receptor ligand activity	0.021	molecular function regulator	0.026	identical protein binding	3.304e-4
hormone activity	0.019	molecular transducer activity	5.788e-9	molecular function regulator	0.039
hyaluronic acid binding	0.012	signaling receptor activity	5.788e-9	signaling receptor binding	1.639e-10
Cellular Component					
extracellular region	0.003	cell periphery	2.522e-9	cellular anatomical entity	1.316e-4
extracellular space	0.010	plasma membrane	1.668e-4	membrane	1.982e-8
endoplasmic reticulum protein-containing complex	0.006	intrinsic component of membrane	0.009	cell periphery	1.000e-24
endoplasmic reticulum lumen	0.014	integral component of membrane	0.010	plasma membrane	1.496e-20
smooth endoplasmic reticulum	0.010	extracellular region	1.000e-24	intrinsic component of membrane	1.091e-6

Table S3: Top 30 KEGG pathways of differentially expressed genes in the ischemic mouse brain at pre, and post-stroke days 1 and 3.

Pre				*Day 1*				*Day 3*			
**ID**	**Pathway**	**GeneRatio**	**p.adjust**	**ID**	**Pathway**	**GeneRatio**	**p.adjust**	**ID**	**Pathway**	**GeneRatio**	**p.adjust**
mmu05022	Pathways of neurodegeneration - multiple diseases	183/1656	3.01E-24	mmu05022	Pathways of neurodegeneration - multiple diseases	147/1626	7.96E-11	mmu05022	Pathways of neurodegeneration - multiple diseases	270/3179	2.95E-21
mmu05010	Alzheimer disease	152/1656	2.42E-21	mmu05010	Alzheimer disease	120/1626	5.671E-09	mmu05010	Alzheimer disease	227/3179	2.30E-20
mmu05014	Amyotrophic lateral sclerosis	140/1656	8.35E-18	mmu05171	Coronavirus disease - COVID-19	111/1626	1.73E-20	mmu05014	Amyotrophic lateral sclerosis	210/3179	4.20E-16
mmu05016	Huntington disease	124/1656	7.30E-19	mmu05014	Amyotrophic lateral sclerosis	99/1626	0.0001565	mmu05016	Huntington disease	196/3179	9.00E-24
mmu05012	Parkinson disease	118/1656	2.42E-21	mmu05016	Huntington disease	98/1626	2.607E-08	mmu05012	Parkinson disease	165/3179	7.44E-18
mmu05020	Prion disease	114/1656	8.35E-19	mmu05020	Prion disease	92/1626	5.612E-09	mmu05020	Prion disease	161/3179	3.34E-15
mmu05171	Coronavirus disease - COVID-19	95/1656	1.88E-12	mmu05012	Parkinson disease	89/1626	2.085E-08	mmu05165	Human papillomavirus infection	158/3179	0.00239
mmu03010	Ribosome	91/1656	3.18E-21	mmu04010	MAPK signaling pathway	87/1626	1.092E-05	mmu05171	Coronavirus disease - COVID-19	147/3179	1.61E-13
mmu05165	Human papillomavirus infection	85/1656	0.0312858	mmu05165	Human papillomavirus infection	87/1626	0.009166	mmu04151	PI3K-Akt signaling pathway	147/3179	0.03543
mmu04714	Thermogenesis	83/1656	3.54E-09	mmu03010	Ribosome	79/1626	6.43E-14	mmu05132	Salmonella infection	146/3179	4.68E-12
mmu04151	PI3K-Akt signaling pathway	83/1656	0.0438969	mmu05205	Proteoglycans in cancer	71/1626	2.261E-07	mmu04144	Endocytosis	145/3179	1.2E-08
mmu05415	Diabetic cardiomyopathy	77/1656	6.67E-09	mmu04024	cAMP signaling pathway	69/1626	1.396E-05	mmu04714	Thermogenesis	139/3179	2.36E-13
mmu05208	Chemical carcinogenesis - reactive oxygen species	77/1656	8.57E-08	mmu05132	Salmonella infection	68/1626	0.0018514	mmu04010	MAPK signaling pathway	136/3179	0.00034
mmu04144	Endocytosis	75/1656	0.0008963	mmu04015	Rap1 signaling pathway	67/1626	2.058E-05	mmu05163	Human cytomegalovirus infection	130/3179	2.6E-06
mmu04010	MAPK signaling pathway	70/1656	0.0384744	mmu04020	Calcium signaling pathway	67/1626	0.0007936	mmu05208	Chemical carcinogenesis - reactive oxygen species	126/3179	9E-10
mmu04723	Retrograde endocannabinoid signaling	67/1656	1.42E-12	mmu05017	Spinocerebellar ataxia	64/1626	5.70E-12	mmu05170	Human immunodeficiency virus 1 infection	123/3179	2.8E-06
mmu04020	Calcium signaling pathway	67/1656	0.0013186	mmu05163	Human cytomegalovirus infection	62/1626	0.0238932	mmu05415	Diabetic cardiomyopathy	120/3179	1.9E-09
mmu05017	Spinocerebellar ataxia	66/1656	3.32E-13	mmu04510	Focal adhesion	61/1626	0.0001494	mmu04360	Axon guidance	116/3179	8.62E-14
mmu05163	Human cytomegalovirus infection	66/1656	0.0095503	mmu04014	Ras signaling pathway	61/1626	0.0067116	mmu04014	Ras signaling pathway	113/3179	0.0002

mmu05132	Salmonella infection	65/1656	0.0107893	mmu04062	Chemokine signaling pathway	60/1626	7.089E-05	mmu03010	Ribosome	112/3179	2.04E-12
mmu04810	Regulation of actin cytoskeleton	64/1656	0.0005773	mmu05417	Lipid and atherosclerosis	60/1626	0.001743	mmu04020	Calcium signaling pathway	111/3179	0.00129
mmu04024	cAMP signaling pathway	63/1656	0.0009535	mmu05169	Epstein-Barr virus infection	60/1626	0.0070882	mmu05166	Human T-cell leukemia virus 1 infection	108/3179	0.01626
mmu04932	Non-alcoholic fatty liver disease	62/1656	6.69E-09	mmu04723	Retrograde endocannabinoid signaling	59/1626	1.606E-08	mmu04510	Focal adhesion	107/3179	1.6E-06
mmu04510	Focal adhesion	61/1656	0.0002427	mmu05415	Diabetic cardiomyopathy	59/1626	0.0016484	mmu04015	Rap1 signaling pathway	107/3179	4.6E-05
mmu04141	Protein processing in endoplasmic reticulum	60/1656	2.83E-06	mmu04261	Adrenergic signaling in cardiomyocytes	58/1626	1.031E-07	mmu04024	cAMP signaling pathway	107/3179	0.00018
mmu05205	Proteoglycans in cancer	60/1656	0.0007546	mmu05208	Chemical carcinogenesis - reactive oxygen species	58/1626	0.007115	mmu04810	Regulation of actin cytoskeleton	107/3179	0.00018
mmu04360	Axon guidance	59/1656	3.71E-05	mmu05170	Human immunodeficiency virus 1 infection	57/1626	0.0397358	mmu05417	Lipid and atherosclerosis	106/3179	0.00013
mmu04014	Ras signaling pathway	59/1656	0.0247004	mmu04621	NOD-like receptor signaling pathway	55/1626	0.0111195	mmu04062	Chemokine signaling pathway	101/3179	6.1E-06
mmu03040	Spliceosome	58/1656	5.97E-10	mmu05207	Chemical carcinogenesis - receptor activation	55/1626	0.0274099	mmu05169	Epstein-Barr virus infection	101/3179	0.01419
mmu00190	Oxidative phosphorylation	57/1656	2.70E-09	mmu04714	Thermogenesis	55/1626	0.041677	mmu04723	Retrograde endocannabinoid signaling	98/3179	4.47E-13
